# Foods and Their Calorific Value

**Published:** 1911-10

**Authors:** G. Bachmann

**Affiliations:** Professor of Physiology, Atlanta College of Physicians and Surgeons


### Journal-Record of Medicine

Successor to Atlanta Medical and Surgical Journal, Established 1855, and Southern Medical Record, Established 1870.

**OWNED BY THE ATLANTA MEDICAL JOURNAL CO.**

**Published Monthly**

*Official Organ Fulton County Medical Society, State Examining Board, Presbyterian Hospital, Atlanta, Birmingham and Atlantic Railroad Surgeons' Association, Chattahoochee Valley Medical and Surgical Association, Etc.*

EDGAR G. BALLENGER, M. D., Editor.
BERNARD WOLFF, M. D., Supervising Editor.
A. W. STIRLING, M. D., C. M., D. P. H., J. S. HURT, B. Ph., M. D.
GEO. M. NILES, M. D., W. J. LOVE, M. D., (Ala.); Associate Editors.
E. W. ALLEN, Business Manager.

COLLABORATORS

Dr. W. F. WESTMORLAND, General Surgery.
F. W. McRAE, M. D., Abdominal Surgery.
H. F. HARRIS, M. D., Pathology and Bacteriology.
MICHAEL HOKE, M. D., Orthopedic Surgery.
CYRUS W. STRICKLER, M. D., Legal Medicine and Medical Legislation.
E. C. DAVIS, A. B., M. D., Obstetrics.
E. G. JONES, A. B., M. D., Gynecology.
R. T. DORSEY, Jr., B. S. M. D., Medicine.
L. M. GAINES, A. B., M. D., Internal Medicine.
J. N. LeCONTE, M. D., Disease of the Stomach and Intestines.
L. B. CLARKE, M. D., Pediatrics.
EDGAR PAULIN, M. D., Opsonic Medicine.
THEODORE TOEPEL, M. D., Mechano Therapy.
R. R. DALY, M. D., Medical Society.
A. W. STIRLING, M. D., etc., Diseases of the Eye, Ear, Nose and Throat.
BERNARD WOLFF, M. D., Diseases of the Skin.
E. G. BALLENGER, M. D., Diseases of the Genito-Urinary Organs.

VOL. LVIII. OCTOBER 1911 No. 7.

### FOODS AND THEIR CALORIFIC VALUE.

BY G. BACHMANN, M. D.

*Professor of Physiology, Atlanta College of Physicians and Surgeons.*

Numerous experiments have established the fact that an adult under ordinary circumstances loses daily, about

2,500 grams of $H_2O$
25 grams of inorganic salts,
280 grams of Carbon ($CO_2$ and urea),
18 grams of nitrogen (urea, uric acid, etc.); and that he utilizes a certain amount of energy, which expressed in heat units, equals about 2,600 calories.

These daily losses must be replaced, and the substances which are used for this purpose are known as foods. We may, therefore, define a food as any substance which is capable of being utilized for the growth and repair of our tissues, and which, when it combines with oxygen, yields energy to the body. As an organism does not live on the food which has just been ingested and absorbed, but on its reserve material derived from the food, it follows that a food; to deserve that name, must be able to contribute directly to this reserve material. For this reason, though alcohol can be oxidized by the body tissues and yield a certain amount of energy, it cannot be classified as a food.

The inorganic food principles are oxygen, water and inorganic salts.

About one-third of the water necessary for the maintenance of our nutrition is taken with the food, as soups and beverages,—which leaves from three to four glasses to be taken as "drinking water" daily.

The organic food principles are carbohydrates, fats and proteins. The most important carbohydrates found in our daily food are starch, cane-sugar, milk-sugar, dextrose and glycogen. While cellulose is largely digested by herbivorous animals, this is not the case in man; however, it plays an important part in digestion, in that it mechanically stimulates the intestines to peristaltic movements and thereby prevents constipation. The importance of cellulose in this respect may be demonstrated by feeding rabbits on food free from cellulose; they inevitably die from volvulus or enteritis.

The amount of carbohydrate food needed daily is difficult of estimation for the reason that it can be replaced by fat. A man doing moderate physical labor ingests from 300 to 800 grams of carbohydrates. Atwater places the amount at 400 grams.

All food stuffs contain fat in varying proportions. This food principle is, as in the case of the carbohydrates, oxidized in the body for the most part. A relatively small proportion is utilized in the formation of the tissues.

The amount of fat required daily by a healthy adult varies in accordance with the quantity of carbohydrates entering in the diet; it is usually from 50 to 120 grams.

Protein occupies a unique place in our dietary in that no other food principle can act as a substitute for it. None of the higher animals can be fed exclusively on carbohydrates and on fats for any length of time. Dogs fed in this manner die in from 30 to 36 days. The result might be different in herbivorous animals, but it serves to illustrate the important part which protein plays in our nutrition. That protein food is necessary may be inferred also from the fact that the essential part of all protoplasm is protein in character and that nearly all the body liquids contain a high percentage of this substance. Furthermore, there is eliminated from the body from 18 to 20 Gm. of nitrogen daily. The only way in which this loss can be replaced is through the ingestion of protein.

What is the minimum quantity of protein which can be ingested and yet be consistent with the maintenance of nutritive equilibrium? This is a question of some importance, not only from an economic, but likewise from a hygienic point of view. The habitual eating of protein foods in excess is inevitably attended by various disorders of nutrition with which all clinicians are familiar.

The amount of protein which the average adult should obtain daily is usually stated to be 120 Gm., or better, 1.5 Gm. per kilogram of body weight. According to Lapicque, the necessary minimum should be 1 gram of protein for every kilogram of body weight. Chittenden has reduced this already small amount still more, namely, 0.75 to 0.8 gram of protein per kilogram of body weight. At this rate an individual ought not to ingest more than from 30 to 50 gms. of protein per day. This, however, apart from a few exceptions is not the experience of mankind; it is noteworthy that the exceptions to a high protein level, those which approach the figures recommended by Chittenden, are found among physically inferior people, who moreover, have a low power of resistance to disease, kidney diseases not excepted.

Mankind, under the guidance of normal appetite and the control of natural selection, has adopted very uniformly the relatively high protein level of from 90 to 100 grams per day. This fact in itself is so impressive and its importance so universally recognized that a certain physician abroad wrote that if Chittenden would come over and try his protein-poor diet on himself, he would not only pay for the food, but likewise defray all funeral expenses. There can be no doubt, however, of the accuracy of Chittenden's experiments, and some of them were continued over such long periods of time (1 year and over,) that the demonstration of the possibility of remaining in nitrogen equilibrim on 0.75-0.8 gm. of protein per kilogram of body weight, is ample. Why is it then that the vigorous races have naturally chosen a higher protein level?

Abderhalden has offered a very plausible explanation based on our knowledge of the chemical structure of proteins.

The molecule of all proteins is formed of the union of different amino-acids; it follows that the living tissue cannot repair itself unless it receive the same kinds of amino-acids as those which enter into its composition. In general, these amino-acids can better be obtained from animal protein than from vegetable protein. Let us take for example the reconstruction of globin, a globulin found in hemoglobin. Globin contains 11% of histidin (a monamino-acid.) Let us suppose that only bread were available as a food to reconstruct globin. Bread contains a protein known as gliadin which has only 1.7% of histidin. Since globin contains 11% of histidin and gliadin only 1.7% there will be needed 100x11÷1.7=647 gm. of gliadin for every 100 gm. of globin. But gliadin conains also from 31-36% of glutaminic acid, so that 647 gm. of gliadin would bring into the organism more than 200 gm. of glutaminic acid, which would be practically useless for reconstructive purposes since 100 gm. of globin contains only 1.73 gm. of this acid.

It is obvious, therefore, that the reconstruction of the protein matter of a tissue can take place economically, only when the proteins ingested contain the kinds of amino-acids found in the tissue and when these are present in nearly the same proportions as in the tissue to be repaired. As stated before, proteins of animal origin meet these conditions more closely than the vegetable proteins.

Food, then, is needed,—first, to repair the tissues, and second, to supply the organism with the energy which appears mainly as heat and mechanical work.

The first need is met especially by protein which supplies the tissues with the amino-acids necessary for their repair. During the period of growth of the body, proteins likewise supply the necessary material. If protein is taken in excess of that required for the replacement of the wear and tear of the tissues, or of their further growth, it is deprived of its amidogen group (in other words, deaminized) and its nitrogen appears in the urine as urea. The deaminized remainder is oxidized in the body and furnishes a certain quota of energy. The energy which the body needs can be supplied much better by means of carbohydrates and fats, more especially by means of carbohydrates.

In order to estimate with mathematical precision the amount of energy which the body spends, and which therefore must be replaced, a definite unit of measurement had to be adopted; this unit of measurement is the calorie. A calorie may be defined as the amount of heat which is necessary to raise the temperature of one kilogram of water 1° C.

As the carbohydrates and the fats can be oxidized completely to CO2 & H2o, inside as well as outside the body, it is only necessary to oxidize in a calorimeter a known quantity of the substance under examination, say 1 gm., and to multiply the number of calories thus developed by the quantity of the substance ingested daily, expressed in grams. The same results are obtained by allowing the carbohydrates or fats to be oxidized within the body at rest and collecting the heat which escapes; the difference of 0.1-0.2 less which may be found is due to the expenditure of mechanical energy by the heart and the respiratory muscles. These methods enable us to estimate the energy yielded to the body by carbohydrates and fats.

In the case of protein the results of oxidation outside and inside the body are different.

The complete oxidation of protein as it takes place outside yields $CO_2$, $H_2O$ and N, whereas within the body the results of oxidation are $CO_2$, $H_2O$ and urea. It is therefore necessary in estimating the heat value of protein to deduct from the total heat value that of the urea formed during oxidation.

Accordingly, the physiological heat value, or calorific value, of the important food principles is:

| | |
|---|---|
| For 1 gram of carbohydrate | 4.1 Cal. |
| For 1 gram of fat | 9.3 Cal. |
| For 1 gram of protein | 4.1 Cal. |

When the body is at absolute rest and is only necessary to replace the daily losses incidental to the performance of the functions of nutrition, the diet should contain, approximately:

400 gm. of carbohydrates.

50 gm. of fat.

70 gm. of protein.

this diet scale is in reality between 2,000 and 2,200 calories.

Owing to unavoidable loss during digestion, the heat value of this diet scale is in reality between 2,000 and 2,200 calories.

If, however, the individual has to perform mechanical work the expenditure of calories will rise in accordance with the intensity of muscular action.

Thus, with work of low intensity it will be 2,500 Cal.

With work of medium intensity it will be 2,868 Cal.

With work of high intensity it will be 3,362 Cal.

The consumption of oxygen rises, of course, with the intensity of the oxidative processes.

### Intestinal Digestion.

There remains to say a few words on digestion as it takes place in the intestines.

It is especially in the upper part of the small intestines that the chemical phenomena inaugurated in the stomach are continued and completed with the aim of transforming the food principles into substances fit for absorption. Here the food principles, more or less or not at all modified by the gastric juice, come in contact with the products of the secretory activity of the glands of Brunner and of Lieberkuhn, as well as, with the pancreatic juice and the bile. The reactions which follow are necessarily complex, for these various secretions do not act separately but simultaneously, and while the physiologist has been forced to study their action separately, this mode of study has led to erroneous conceptions for the reason, that in this way the cynergetic action of these secretions on each other escaped detection until quite recently.

In order to better understand the action of these secretions, let us recall the substances which pass out of the stomach into the small intestine. This material is the so-called chyme, and when first forced out of the stomach, is, of course, acid in reaction. It passes out of the stomach in small quantities at a time so that the contents of the intestines have no difficulty in neutralizing the acid and rendering the chyme faintly alkaline in reaction. Chyme consists of water, inorganic salts, starch, dextrin, maltose, lactose, saccharose, dextrose, cellulose, native proteins, acid proteins, proteoses, peptones, fat.

The action of the pancreatic juice depends on the presence in it of at least three enzymes. The amylopsin causes hydrolysis of the starch, converting it into dextrin and maltose; as there is also some maltase in the pancreatic juice, some of the maltase formed by the action of amylopsin is split into two molecules of dextrose. Steapsin or lipase acts on the fats. By a process of hydrolysis, the neutral fats are split into their corresponding fatty acids and glycerine. This constitutes what is known as saponification. As there are alkaline bases present in the intestinal contents the fatty acids combine with these, the results being the formation of soaps. The energy set free during the cleavage of the fats causes the remaining neutral fat to become finely divided. The tiny globules remain reparated and suspended in the soap solution. This process is know as emulsification; it is merely an incident, and not an essential part in the digestion of fat. However, owing to the greater surface of fat exposed, emulsification may aid materially in the further action of lipase.

Trypsin acts on the protein part of the food which has escaped gastric digestion. Trypsin hydrolizes protein much more completely and rapidly than does pepsin. However, the preliminary action of pepsin seems to be favorable to the action of trypsin. In the cleavage of protein, this food principle passes through the stages of alkali protein, proteoses, then peptones; until recently, this was thought to be the end of tryptic digestion, inasmuch as peptones being highly diffusible, could readily be absorbed. We now know, that the cleavage of the protein molecule goes much farther, the peptones being split into a number of simpler products known as poly-peptids and amino-acids These substances no longer give the biuret reaction and are crystallizable bodies. It is suspected that the final cleavage of the peptones is caused, not by trypsin, but by another ferment called erepsin, also found in the intestinal juice. The action of the pancreatice ferments is noticeably increased when in combination with the intestinal juice. Indeed, in the case of trypsin, it is necessary that its antecedent the so-called trypsinogen be first activated by another ferment found in the intestinal juice and known as enterokinase.

The intestinal juice contains at least five ferments: the enterokinase and erepsin already mentioned, invertase, maltase and lactase. Invertase, sometimes called sucrase, inverts cane-sugar into equal numbers of molecules of dextrose and levulose; maltase splits maltose into two molecules of dextrose, and lactase splits milk-sugar into dextrose and galactose.

The small intestine furthermore produces a hormone, the so-called secretin, which, when activated by hydrochloric acid, excites the flow of the pancreatic juice and of the bile.

